# Primary cilia and lipid raft dynamics

**DOI:** 10.1098/rsob.210130

**Published:** 2021-08-25

**Authors:** Yuhei Nishimura, Daishi Yamakawa, Katsunori Uchida, Takashi Shiromizu, Masatoshi Watanabe, Masaki Inagaki

**Affiliations:** ^1^ Department of Integrative Pharmacology, Mie University Graduate School of Medicine, Tsu, Mie, Japan; ^2^ Department of Physiology, Mie University Graduate School of Medicine, Tsu, Mie, Japan; ^3^ Department of Oncologic Pathology, Mie University Graduate School of Medicine, Tsu, Mie, Japan

**Keywords:** primary cilia, lipid raft dynamics, Akt, adipogenesis, cancer

## Abstract

Primary cilia, antenna-like structures of the plasma membrane, detect various extracellular cues and transduce signals into the cell to regulate a wide range of functions. Lipid rafts, plasma membrane microdomains enriched in cholesterol, sphingolipids and specific proteins, are also signalling hubs involved in a myriad of physiological functions. Although impairment of primary cilia and lipid rafts is associated with various diseases, the relationship between primary cilia and lipid rafts is poorly understood. Here, we review a newly discovered interaction between primary cilia and lipid raft dynamics that occurs during Akt signalling in adipogenesis. We also discuss the relationship between primary cilia and lipid raft-mediated Akt signalling in cancer biology. This review provides a novel perspective on primary cilia in the regulation of lipid raft dynamics.

## Graphical abstract

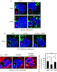

## 1. Introduction

Primary cilia are non-motile, 1–10 µm long antenna-like structures observed in a variety of vertebrate cells. Primary cilia sense extracellular signals, such as biochemical and mechanical stimuli, and transduce these signals into the cell to regulate functions such as differentiation and proliferation [1–11]. Primary cilia convey a wide range of signals, including the signalling pathways of hedgehog, receptor tyrosine kinases (RTKs) and G protein-coupled receptors such as lysophosphatidic acid receptors [12,13]. Thus, the structure and function of primary cilia are appropriately regulated in normal physiology and their dysregulation is associated with various human diseases [5,9,14,15].

Lipid rafts are nanoscale membrane microdomains where lipids, such as cholesterol and sphingolipids, are enriched [16–20]. Specific proteins assemble with these lipids and mediate signalling in response to intra- or extracellular stimuli [21]. Therefore, lipid rafts work as compartmentalized platforms to regulate signalling for multiple cellular functions, including proliferation, differentiation and apoptosis [22,23]. The serine/threonine kinase, Akt, is a representative signalling protein associated with lipid rafts [24–26]. For example, Akt is activated by the insulin and insulin-like growth factor 1 (IGF1) pathways, which is facilitated by lipid rafts [22,23]. In the presence of these ligands, the insulin receptor (IR) and the IGF1 receptor (IGF1R) phosphorylate IR substrate, which activates phosphatidylinositol 3-kinase (PI3K). Activated PI3K produces phosphatidylinositol 3,4,5-triphosphate (PIP3) by phosphorylation of phosphatidylinositol 4,5-bisphosphate (PIP2). PIP3 binds to the pleckstrin homology domain of Akt, promoting the binding of Akt to phosphatidylinositol-dependent protein 1 (PDK1) and mammalian target of rapamycin complex (mTORC)2, which phosphorylate Thr308 and Ser473 of Akt, respectively. All these components are located in lipid rafts, increasing the efficiency of Akt activation. Phosphorylated Akt regulates downstream signalling proteins in fundamental cellular functions, whereas over-activation of lipid raft-mediated Akt signalling is associated with various metabolic diseases [22,23].

Although both primary cilia and lipid rafts act as signalling hubs, their relationship has been poorly understood. In this review, we explain a novel role of primary cilia on certain types of lipid raft dynamics related to IR–Akt signalling upon adipogenic stimuli in mouse adipose progenitor cells (APs) and mouse mesenchymal progenitor C3H10T1/2 cells [27]. We also discuss potential therapeutic approaches for obesity and cancer targeting lipid raft dynamics and primary cilia.

## 2. Lipid rafts in the regulation of RTKs signalling

RTKs, such as IR, IGF1R, epidermal growth factor receptor (EGFR), fibroblast growth factor receptor (FGFR) and platelet-derived growth factor receptor (PDGFR), are transmembrane proteins that are activated by ligand binding, which usually causes dimerization and/or oligomerization of the receptors followed by trans-autophosphorylation of multiple tyrosine residues [28]. The phosphorylated RTKs activate various downstream signalling pathways, such as the PI3K–Akt cascade, the mitogen-activated protein kinase cascade and the Janus kinase-signal transducer and activator of transcription cascade [28]. Some of the RTKs signallings events can be modulated by lipid rafts [29–31]. The mechanisms include positive or negative regulation of RTK trans-phosphorylation by clustering within the lipid rafts and concentration or exclusion of signalling molecules downstream of RTKs within lipid rafts [29,30,32].

Lipid rafts can be grouped based on the main components [30,31,33–35]. Caveolins and flotillins are representative scaffold proteins in lipid rafts [31,33]. Caveolin 1 (CAV1) inserts into the inner leaflet of the membrane via a hairpin loop with the N- and C-termini exposed to the cytoplasm [36]. The N-terminal domain facilitates the formation of homo-oligomers [37] and interaction with other proteins that have a binding motif to CAV1 (e.g. IR) [36,38]. In the presence of Caveolae-associated protein 1, CAV1 can make caveola, structurally a unique lipid raft that is characterized by flask-shaped invagination of the plasma membrane [39]. CAV1 accelerated IR–Akt signalling in the differentiation of preadipocytes and the energy metabolism of adipocytes [27,40]. By contrast, CAV1 inhibited the autophosphorylation of EGFR and PDGFR in epidermoid carcinoma and fibroblasts, respectively [41,42]. Flotillins are two homologous proteins, flotillin 1 and flotillin 2 [43]. Flotillin 1 positively regulated the phosphorylation of EGFR and a substate of FGFR in HeLa cells [44,45]. The correlation between the composition and the function of lipid rafts, however, remains elusive [17].

## 3. Primary cilia regulate lipid raft dynamics related to IR–Akt signalling in adipogenesis

Adipogenesis plays an important and complex role in metabolic health [46]. Differentiation from preadipocytes to adipocytes is an adaptive response to nutritional overload [47]. Of note, adipogenesis can be detrimental depending on the context. For example, adipogenesis in the skeletal muscle induced by over-nutrition impairs wound healing [48]. Adipogenesis in bone marrow caused by ageing is associated with an increased risk of bone fracture [49]. Primary cilia are involved in this physiological and pathological adipogenesis. Differentiation from preadipocytes to adipocytes is stimulated by adipogenic signalling through activation of IR/IGF1R located at the ciliary base [27,50]. Upon adipogenic stimulation, IR/IGF1R observed at the region surrounding the ciliary base stimulate the PI3K–Akt pathway [27,51,52]. The activated Akt increases the expression of PPARγ, the master regulator of adipogenesis, by inhibiting the translocation of FOXO1, a key transcriptional suppressor in adipogenesis, to the nucleus of preadipocytes [53,54]. Impairment of IR signalling was associated with the metabolic dysfunction in Bardet–Biedl syndrome, a rare autosomal recessive ciliopathy [55–57]. Primary cilia also regulate adipogenesis through omega-3 fatty acid-activated FFAR4/GPR120 and desert hedgehog-activated smoothened [58,59]. However, the relationship between primary cilia and lipid rafts in the regulation of Akt signalling remains largely unknown [26,60].

**Figure 1.**
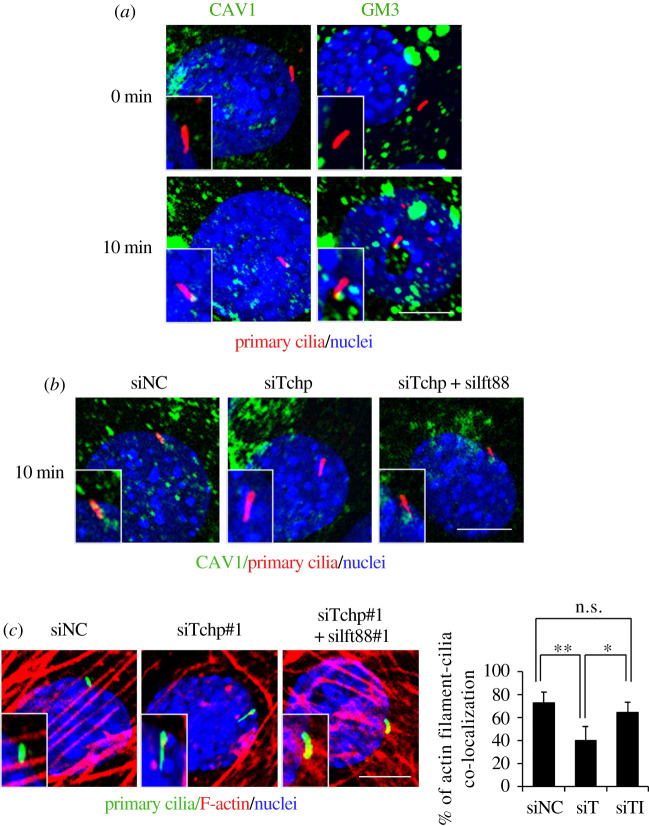
Elongated primary cilia in mouse mesenchymal progenitor C3H10T1/2 cells affect lipid raft dynamics at the ciliary base upon adipogenic stimuli and the formation of actin filaments beneath the cilia. (*a*) Immunocytological analysis of C3H10T1/2 cells before (0 min) and after (10 min) adipogenic stimulation. The lipid rafts were stained using anti-Caveolin 1 (CAV1) and anti-GM3 antibodies. The primary cilia and nuclei were stained using an anti-acetylated tubulin or anti-Arl13b antibody and Hoechst33342, respectively. (*b*) Immunocytological analysis of CAV1, primary cilia and nuclei in C3H10T1/2 cells treated with non-targeting control siRNA (siNC), siRNA for trichoplein (siTchp) or siRNAs for trichoplein and Ift88 (siTchp + siIft88). (*c*) Immunocytological analysis of primary cilia, actin filaments and nuclei in C3H10T1/2 cells treated with siNC, siTchp or siTchp + siIft88. Actin filaments were stained using fluorescent phalloidin. *p < 0.05; **p < 0.01.

We have shown that trichoplein (TCHP), a centriolar protein originally identified as a keratin-binding protein, is a key activator of aurora A kinase (AURKA) at centrioles and that knockdown (KD) of TCHP causes elongation of primary cilia in human retinal pigmental epithelium cells by suppressing the inhibitory effect of AURKA in ciliogenesis [7,61–66]. We recently found that *Tchp* knockout (KO) mice show elongated cilia in APs, reduced body fat and smaller adipocytes than wild-type mice under a high-fat diet [27]. In APs from wild-type mice, CAV1- or ganglioside GM3-positive lipid rafts moved to the ciliary base upon adipogenic stimulation [27] (figure 1*a*), while GM1 and flotillin-2, other markers of lipid rafts, did not accumulate around the primary cilia [27]. In APs from *Tchp* KO mice, all four types of lipid rafts did not appear near the ciliary base upon adipogenic stimulation.

We also found that lipid raft dynamics were impaired in C3H10T1/2 cells with *Tchp* KD, in which the primary cilia were elongated, but not in C3H10T1/2 cells with double KD of *Tchp* and *Ift88*, in which the elongation of primary cilia caused by *Tchp* KD was suppressed by concomitant KD of *Ift88*, resulting in primary cilia being of similar length to that of control C3H10T1/2 cells [27] (figure 1*b*). The phosphorylation of Akt in mouse APs upon adipogenesis and the differentiation into adipocytes were also suppressed by *Tchp* KD but not by double *Tchp* and *Ift88* KD [27]. The correlation between the impaired accumulation of CAV1-positive lipid rafts and the decreased phosphorylation of Akt at the ciliary base where IRs are localized is consistent with previous studies demonstrating that CAV1 positively modulates IR/IGF1R-Akt signalling in adipogenesis [67,68].

The finding that primary cilia regulate Akt signalling in adipogenesis by modulating lipid raft dynamics provides a novel perspective on the crosstalk between primary cilia and lipid rafts [27]. Co-localization of actin filaments with primary cilia was significantly lower in C3H10T1/2 cells with *Tchp* KD than in control C3H10T1/2 cells or C3H10T1/2 cells with double *Tchp* and *Ift88* KD (figure 1*c*). CAV1 clusters are tethered to cortical actin filaments through interaction with filamin, an actin-binding protein [69]. These results indicate that the elongated cilia in C3H10T1/2 cells caused by *Tchp* KD may affect lipid raft dynamics through impairment of the actin-based transport system. The mechanisms by which primary cilia regulate lipid raft dynamics warrant further examination.

## 4. Primary cilia and lipid raft-mediated Akt signalling in cancer biology

Lipid raft-mediated Akt signalling is over-activated in various types of cancer, including prostate cancer and melanoma [26,70–73]. Phosphatase and tensin homologue deleted from chromosome 10 (PTEN) inhibits Akt activity through dephosphorylation of PIP3 to PIP2 [74]. Protein phosphatase 2A (PP2A) also inhibits Akt activity through dephosphorylation of Thr308 and Ser473 of Akt [75]. The loss of PTEN is frequently observed in both prostate cancer and melanoma [76,77]. Impairment of PP2A is also reported in these cancers [78,79]. Lipid raft-mediated Akt signalling is over-activated by impairment of PTEN or PP2A [80–82]. Sterol regulatory element-binding protein 1 (SREBP1) is positively regulated by the PI3K–Akt–mTORC1 pathway [83,84]. In fact, the activity of SREBP1 is increased in both prostate cancer and melanoma [85]. Activation of SREBP1 also stimulates PI3K–Akt signalling [84,86]. Various therapeutic approaches for cancer have been developed that target PI3K, PTEN, Akt, PP2A, or the organization of these molecules in lipid rafts [23,71,73,87–93].

**Figure 2.**
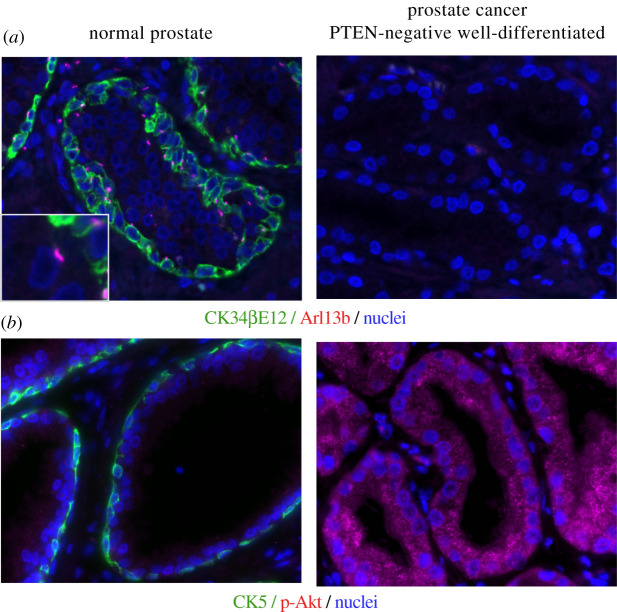
Loss of primary cilia and increase of phosphorylated Akt in PTEN-negative and well-differentiated prostate cancer. Immunohistological analysis of basal cell, primary cilia and nuclei (*a*), or basal cell, phosphorylated Akt and nuclei (*b*), in normal prostate and PTEN-negative well-differentiated prostate cancer. Basal cells were stained using an anti-CK34βE12 antibody to detect cytokeratin (CK) 1/5/10/14 (*a*) or an anti-CK5 antibody (*b*). The primary cilia and nuclei were stained using an anti-Arl13b antibody and DAPI, respectively. Phosphorylated Akt was stained using an anti-phosphorylated Akt antibody.

Interestingly, primary cilia are often lost in prostate cancers and melanoma [94–98] (figure 2). Knockdown of SREBP1 restored primary cilia to A375 melanoma cells [99]. Knockdown of fatty acid synthase, a transcriptional target of SREBP1, also restored primary cilia to the prostate cancer cell line, LNCaP [100]. Treatment with rapamycin, an mTORC1 inhibitor, restored the impaired ciliogenesis of prostate cancer cell lines (PC3 and DU145) and inhibited their proliferation [101], which is consistent with studies demonstrating that primary cilia work as a brake on cell proliferation [6–10,63,64,66,102].

## 5. Therapeutic approaches targeting lipid raft dynamics through primary cilia

Lipid rafts have attracted attention as a novel target in cancer therapy [23,71,73,103,104]. For example, T0901317, a liver X receptor agonist, suppressed lipid raft-mediated Akt signalling affecting the localization and expression of flotillin-2 in a prostate cancer cell line, LNCaP, and a breast cancer cell line, MCF-7 [105,106]. Rh2, a major bioactive constituent of *Panax ginseng*, a traditional medicine, inhibited lipid raft-mediated Akt signalling, which affected the dynamics of CAV1 and GM1 in an epidermoid carcinoma cell line, A431 [107]. Edelfosin and perifosine, single-chain alkylphospholipids, suppressed the phosphorylation of Akt and the growth of patient-derived xenografts of mantle cell lymphoma [108]. Strategies for improving the efficacy and safety of these therapeutic drugs have been actively developed [109–111]. Statins can also be classified as anti-cancer agents that inhibit lipid raft-mediated Akt signalling [71,73,103,104].

The possibility that primary cilia may take part in lipid raft dynamics [27] provides a novel approach to modulate lipid raft-mediated Akt signalling through targeting primary cilia. Genome-wide screens have identified various ciliary proteins that directly regulate ciliogenesis and the associated proteins that regulate the expression and function of these ciliary proteins [64,66,112–119]. Multi-omics approaches, including lipidomics and glycomics, can also be used to identify novel components involved in the formation of primary cilia and lipid raft-mediated signalling. For example, O-linked β-*N*-acetylglycosaminylation (O-GlcNAcylation) is related to lipid raft-mediated Akt signalling [120,121] and ciliogenesis [122,123], and is associated with obesity, diabetes and cancer [124–126]. Modulation of O-GlcNAcylation may be a novel therapeutic approach to regulate lipid raft dynamics and primary cilia.

AURKA inhibitors, as well as Akt inhibitors, have been developed and evaluated in cancer and obesity [102,127–131]. In mouse preadipocytes, KD of *Aurka*, a downstream target of *Tchp* [63], stimulated elongation of primary cilia and inhibited IR–Akt signalling and adipogenesis [27]. This suggests that forced elongation of primary cilia caused by AURKA inhibition may rectify the dysregulation of cell proliferation and/or differentiation caused by impairment of ciliogenesis in cancer and obesity.

Identification of novel factors involved in ciliogenesis and lipid raft dynamics in specific cell types may provide critical information to understand the pathophysiology of diseases associated with the dysregulation of these signalling pathways, which could lead to the development of novel therapeutic drugs targeting lipid raft dynamics and primary cilia.

## Data accessibility

This article has no additional data.
